# Recent advances in pancreatic α-cell transdifferentiation for diabetes therapy

**DOI:** 10.3389/fimmu.2025.1551372

**Published:** 2025-01-22

**Authors:** Yanjiao Li, Jinyu Zhu, Congyang Yue, Siyuan Song, Limin Tian, Yi Wang

**Affiliations:** ^1^ Department of Pharmacy, Qionglai Hospital of Traditional Chinese Medicine, Chengdu, China; ^2^ Clinical Immunology Translational Medicine Key Laboratory of Sichuan Province, Sichuan Provincial People’s Hospital, University of Electronic Science and Technology of China, Chengdu, Sichuan, China; ^3^ Center for Geriatrics and Endocrinology, Sichuan Provincial People’s Hospital, University of Electronic Science and Technology of China, Chengdu, Sichuan, China; ^4^ Department of Neuroscience, Baylor College of Medicine, Houston, TX, United States; ^5^ Center for Critical Care Medicine, Sichuan Provincial People’s Hospital, University of Electronic Science and Technology of China, Chengdu, Sichuan, China

**Keywords:** diabetes, pancreatic α-cells, β-cell transdifferentiation, transcription factors, insulin secretion, clinical applications, cell stability, regenerative medicine

## Abstract

As the global prevalence of diabetes mellitus rises, traditional treatments like insulin therapy and oral hypoglycemic agents often fail to achieve optimal glycemic control, leading to severe complications. Recent research has focused on replenishing pancreatic β-cells through the transdifferentiation of α-cells, offering a promising therapeutic avenue. This review explores the molecular mechanisms underlying α-cell to β-cell transdifferentiation, emphasizing key transcription factors such as *Dnmt1*, *Arx*, *Pdx1*, *MafA*, and *Nkx6.1*. The potential clinical applications, especially in type 1 and type 2 diabetes characterized by significant β-cell dysfunction, are addressed. Challenges, including low transdifferentiation efficiency, cell stability, and safety concerns, are also included. Future research directions include optimizing molecular pathways, enhancing transdifferentiation efficiency, and ensuring the long-term stability of β-cell identity. Overall, the ability to convert α-cells into β-cells represents a transformative strategy for diabetes treatment, offering hope for more effective and sustainable therapies for patients with severe β-cell loss.

## 1 Introduction

Diabetes mellitus is a metabolic disease characterized by hyperglycemia and ranks among the top ten leading causes of death worldwide (1). Due to defective insulin secretion or impaired biological action, it leads to chronic damage to various tissues, particularly the eyes, kidneys, heart, blood vessels, and nerves, resulting in life-threatening complications (2). According to data released by the International Diabetes Federation (IDF), as of 2021, approximately 537 million people worldwide were living with diabetes. In China, there were about 141 million cases, accounting for a quarter of the global diabetic population. With the improvement of living standards, the prevalence of diabetes continues to rise year by year (3).

Blood glucose levels are the sole criterion for diagnosing diabetes. Individuals with obvious symptoms of “three more and one less” can be diagnosed with one abnormal blood glucose value. Asymptomatic individuals require two abnormal blood glucose readings for diagnosis. For suspected cases, a 75g glucose tolerance test is conducted, and the diagnosis is confirmed by a fasting blood glucose level of ≥7.0 mmol/L and/or a two-hour postprandial blood glucose level of ≥11.1 mmol/L (4). Current treatments focus on maintaining blood glucose levels using exogenous insulin or oral antihyperglycemic drugs. However, these approaches often fail to adequately balance glucose metabolism, leading to hyperglycemic episodes and severe complications (5). The progression of diabetes is closely associated with a reduced number and functional impairment of pancreatic islet β-cells (6).

Studies have shown that inducing the transdifferentiation of α-cells into β-cells can replenish the β-cell population and restore their glucose-regulating function, thereby offering a promising therapeutic strategy for diabetes (7). Research on the transdifferentiation of pancreatic islet α-cells into β-cells provides novel insights and potential treatments for diabetes, particularly in cases of severe β-cell loss (8, 9). This paper reviews the mechanisms underlying α-cell transdifferentiation, recent research progress, and future research directions, aiming to contribute to the development of new therapeutic approaches for diabetes.

## 2 Biological basis of islet α-cell to β-cell transdifferentiation

### 2.1 The composition of islet cells

The pancreas is a complex glandular organ composed of two major cell groups: the endocrine pancreas and the exocrine pancreas, which differ significantly in both function and morphology (10, 11). The exocrine pancreas primarily comprises acinar cells responsible for enzyme secretion, arranged in clusters at the ends of the ductal system. In contrast, the endocrine pancreas contains five distinct hormone-secreting cell types: α-cells, which secrete glucagon; β-cells, which produce insulin; δ-cells, which release somatostatin; ϵ-cells, which secrete ghrelin; and PP-cells, which produce pancreatic polypeptide (12). These hormones play crucial roles in regulating nutrient metabolism and maintaining glucose homeostasis. Notably, α-cells and β-cells are the primary regulators of blood glucose stability, with glucagon and insulin secretion, respectively.

### 2.2 Molecular basis for α-cell to β-cell transdifferentiation

Studies suggest that other endocrine cell types, for example, α-cells, δ-cells can transdifferentiate into β-cells (13). Among these, α-cells share significant developmental similarities with β-cells, providing a biological basis for their reprogramming (14). This makes α-cells the most promising candidates as an alternative source for β-cell replenishment. Firstly, both α-cells and β-cells are located within the endocrine pancreas and maintain a close interrelationship. In rodent pancreatic islets, β-cells constitute approximately 60% of the cell population, while α-cells comprise about 20%. Around 30-40% of β-cells are in direct contact with α-cells. In human pancreatic islets, β-cells account for 50% and α-cells for about 40%, with roughly 70% of β-cells closely associated with α-cells (15). More than 90% of α-cells are in contact with β-cells in both mouse and human islets, underscoring the feasibility of α-to-β cell transdifferentiation for diabetes treatment. Secondly, α-cell proliferation is commonly observed in diabetic animal models and patients, providing a substantial cellular resource for β-cell reprogramming (16). Lastly, studies have reported that a significant reduction in α-cell numbers does not adversely affect glucose metabolism (17). Moreover, glucagon signaling, mediated by α-cells, has been implicated in the progression of diabetes. Therefore, converting α-cells into β-cells may not only replenish β-cell numbers but also mitigate the deleterious effects of glucagon on glycemic control (18).

## 3 Molecular mechanism of islet α-cell to β-cell transdifferentiation

**Figure 1:**
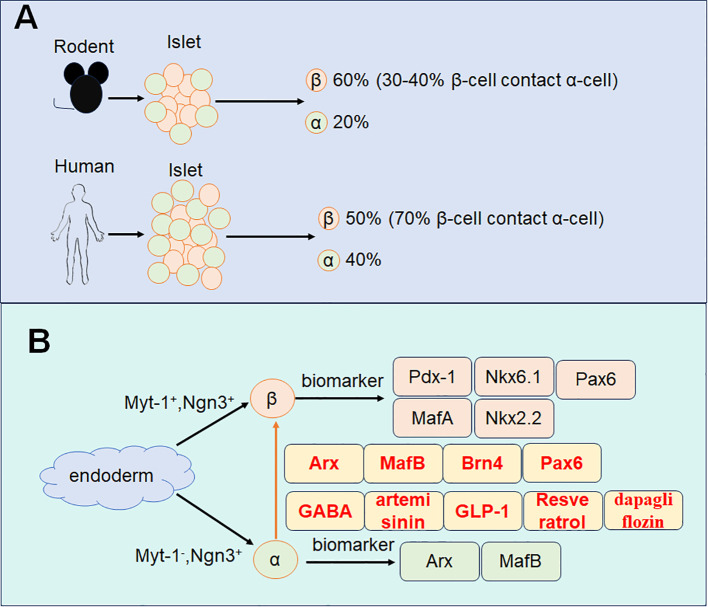
Schematic diagram of α-cell transdifferentiates to β-cell. **(A)** The composition of α-cell and β-cell in rodents and humans. **(B)** The development of α-cell and β-cell with specific marker and the transdifferentiation markers.

The human pancreas originates from the dorsal and ventral regions of the endoderm, giving rise to pancreatic buds at distinct stages of embryonic development (19). These buds consist of multipotent progenitor cells characterized by the expression of pancreatic duodenal homology box protein 1 (*Pdx1*) (20–22). These progenitor cells subsequently differentiate into two distinct lineages: tip cells, which specialize into acinar cells, and bipotent progenitor cells, which differentiate into either ductal or endocrine cells (23) (**Figure 1**).

### 3.1 Key regulators in progenitor cell transdifferentiation to β-cell

Within the dorsal and ventral endoderm, *Pdx1*-positive cells signify the origin of endocrine progenitor cells (24). Neurogenin 3 (*Ngn3*), a pivotal transcription factor, plays an essential role in initiating the differentiation of endocrine progenitors into various endocrine cell types (25, 26). These endocrine progenitor cells exhibit heterogeneity based on the expression of transcription factors such as myelin transcription factor 1 (*Myt1*) and *Ngn3* (27–29). Specifically, *Myt1*-positive and *Ngn3*-positive progenitors are predisposed to develop into β-cells, whereas *Myt1*-negative and *Ngn3*-positive progenitors are more likely to differentiate into α-cells (27).

### 3.2 Biomarkers for α-cell and β-cell

Mature α-cells are characterized by the expression of transcription factors including Aristaless-related homeobox (*Arx)* (30), and *MafB* (31, 32). While β-cells express a distinct set of transcription factors such as *Pdx1* (33), *MafA* (34), *Nkx6.1* (35), NK2 homeobox 2 (*Nkx2.2*) (36), and paired box gene 6 (*Pax6*) (37–39). These distinct transcriptional profiles underscore the molecular divergence between α-cells and β-cells and provide the basis for their unique functions within the endocrine pancreas.

### 3.3 Pathways for the transdifferentiation of α-cell to β-cell

Transdifferentiation of α-cells to β-cells is a key demonstration of endocrine cell plasticity within pancreatic islets, involving a series of intricately regulated molecular mechanisms. This process can be divided into three main stages: α-cell proliferation, the transdifferentiation of α-cells to β-cells, and the stabilization of β-cell identity post-transdifferentiation. A study from 2010 reported that α-cells significantly proliferate in diabetic pancreatic islets in response to extensive β-cell loss (40). This study highlights the inherent plasticity of α-cells under conditions of islet injury or dysfunction, suggesting their ability to respond to the demands of the islet microenvironment by proliferating. This proliferation serves not only as a response to β-cell depletion but also as a preparatory stage for α-cell transdifferentiation into β-cells. The process of α-cell to β-cell transdifferentiation requires tightly coordinated changes in several critical transcription factors.

#### 3.3.1 *Arx*, *MafB*, *Brn4*, and Pax6

The maintenance of α-cell identity depends on transcription factors such as *Arx*, *MafB*, *Brn4*, and *Pax6*, which ensure the stability of the α-cell phenotype by suppressing the activation of β-cell-specific genes (41). Consequently, the initiation of α-cell to β-cell transdifferentiation requires the downregulation of these transcription factors. Among them, *Arx* was the first transcription factor identified as essential for maintaining the α-cell phenotype. Deletion of *Arx* in pancreatic α-cells of diabetic mice has been shown to induce α-cell to β-cell transdifferentiation, normalize blood glucose levels, regenerate β-like cell clusters, and extend lifespan (42). *MafB* and *Brn4* play crucial roles in regulating glucagon production and secretion by binding to the G1 element of the preglucagon gene promoter. Deletion of *MafB* and *Brn4* in mouse α-cells leads to dysregulated glucagon synthesis and secretion (31, 41). *Pax6* is another vital transcription factor in α-cell differentiation. Its functions extend beyond promoting glucagon production to directly or indirectly influencing the transcription and post-transcriptional processing of the glucagon gene (43). After the successful transformation of α-cells into β-cells, it is essential to ensure the stable maintenance of β-cell identity in the transdifferentiated cells, preventing their reversion to α-cells or transformation into other islet cell types. The stabilization of β-cell identity relies on the upregulation of a specific set of transcription factors that enhance the expression of β-cell-specific genes while suppressing the activation of α-cell-characteristic genes. Key transcription factors involved in maintaining the β-cell phenotype include *Pdx1*, *MafA*, *Nkx6.1*, and *Nkx2.2* (44). *PDX1* (also known as insulin initiation factor 1) is a homeodomain-containing transcription factor critical for β-cell function. Studies in mice have shown that deletion of *PDX1* in mature β-cells induces glucose intolerance, underscoring its pivotal role in maintaining β-cell functionality (45). Similarly, low levels of *PDX1* expression have been reported in pancreatic islet β-cells of individuals with type 2 diabetes mellitus, further emphasizing its importance (46). *MAFA*, a member of the MAF family of basic leucine zipper transcription factors, binds specifically to the conserved insulin enhancer element RIPE3b/C1-A2, activating insulin gene expression. Knockdown of *Mafa* in mice results in glucose intolerance and, ultimately, diabetes (47). *Nkx2.2* and *Nkx6.1* are additional transcription factors critical for β-cell development and identity maintenance. *Nkx2.2* is regulated by the highly conserved NK2-specific domain and, in turn, regulates *Nkx6.1* expression (36). *Nkx6.1* not only modulates insulin synthesis-related genes but also inhibits glucagon expression by competing with *Pax6* for the G1 element of the *Gcg* promoter (48). The coordinated upregulation of these transcription factors promotes the expression of β-cell-specific genes while simultaneously suppressing α-cell-associated genes, ensuring the continued stability of β-cell identity after transdifferentiation. A deeper understanding of these molecular mechanisms provides valuable insights into the pathogenesis of diabetes and lays the groundwork for developing innovative therapeutic strategies based on cellular reprogramming.

## 4 Islet cell transdifferentiation in diabetes treatment

### 4.1 Conventional therapy and challenges

For type 1 diabetes, which is characterized by the absolute deficiency of insulin, conventional insulin administration remains the main therapy for it (49). However, These patients will face blood glucose fluctuation and fail to achieve normal glycemia (50). For type 2 diabetes, it is characterized by insulin resistance, therefore, metformin and other drugs should be delivered (51), even if there are obvious adverse effects (52–54). To achieve glucose homeostasis, islet transplantation has been clinically applied. However, there still exist obstacles for it, such as organ shortage, immune rejection, and cytotoxicity to islet β-cell of immunosuppressant (55, 56). Therefore, it is of crucial importance to endogenous regenerate islet β-cell.

### 4.2 Differentiation of stem cell or progenitor cell to islet β-cell

In response to the donor shortage, cell-based therapies have been investigated as alternative treatment options. Early efforts focused on inducing embryonic stem cells (ESCs) to differentiate into β-cells. However, this approach faced ethical concerns and technical limitations, such as the incomplete maturation of ESC-derived β-cells and consistently low expression of key β-cell markers, which restricted its clinical applicability (57, 58). In 2006, Takahashi and Yamanaka introduced a groundbreaking method to generate induced pluripotent stem cells (iPSCs) by reprogramming mouse and human fibroblasts, as well as other adult somatic cells, into pluripotent states using three or four transcription factors. While this method represents a significant advance, the iPSC-derived β-cells are not yet sufficiently mature and often form polyhormonal cells. Additionally, undifferentiated iPSCs carry a risk of forming teratomas (59). Notably, in 2020, Prof. Zeng Yi identified adult mouse pancreatic stem cells capable of differentiating into β-cells and developed protocols to culture these stem cells *in vitro* and induce them to form pancreatic-like organs (60). Subsequently, in 2021, ductal precursor cells were successfully induced to differentiate into β-cells, closely resembling the adult stem cells identified by Prof. Zeng Yi. However, further studies are required to determine whether these two cell types are identical (61). Looking forward, key challenges in this field include enhancing the efficiency of *in vitro* and *in vivo* β-cell proliferation (62, 63), optimizing methods for β-cell storage (64, 65), and improving transportation techniques. Addressing these issues will be critical for advancing β-cell therapy as a viable solution for diabetes treatment.

### 4.3 Potential drugs or peptides to induce the transdifferentiation of α-cell to β-cell

Promoting the transdifferentiation of α-cells to β-cells *in vivo* offers a promising strategy for replenishing β-cell numbers and maintaining adequate insulin secretion. Research has demonstrated that γ-aminobutyric acid (GABA) and artemisinin can facilitate this process by inhibiting the expression of the *Arx* gene in α-cells in both rodents and humans (66). Additionally, GLP-1 (glucagon-like peptide-1) has been shown to induce insulin gene transcription and biosynthesis, enhance glucose-stimulated insulin secretion, promote β-cell proliferation, and prevent β-cell apoptosis, collectively improving glycemic control by maintaining β-cell stability (67).

By linage tracing, their generated recombinant adenovirus expressing GLP-1 (rAd-GLP-1)-treated RIP-CreER;R26-YFP mice, and found that new β-cells are originated from non-β-cells (α-cells). They also observed that by rAd-GLP-1 or exendin-4 treatment, the number of Insulin and glucagon double positive cells were significantly increased both *in vivo* and *in vitro*. Utilizing mouse α-cells (αTC9), resveratrol could induce the expression of transcription factors such as *Pdx1* and *Ins2* in β-cells in a SirT1-dependent manner, thereby promoting the transdifferentiation of α-cells to β-cells (68). Similarly, dapagliflozin, an SGLT2 inhibitor, not only exhibits hypoglycemic effects but also provides direct protection to β-cells and induces α-to-β-cell transdifferentiation through GLP-1 signaling (69). Another study using transgenic mice conditionally expressing transcription factors as Mafa and Pdx1 in the islet β-cell lead to the transdifferentiation of embryonic pan-islet cell Ngn3-positive progenitors and the glucagon-positive α-cell transdifferentiation into β-cells. They observed that Mafa could stimulate the transcription of Pdx1, and thereby inducing β-cell formation from Ngn3^+^ progenitor cells and α-cells from transdifferentiation (70).

For the *in vivo* study, both db/db mice and pancreatic alpha cell lineage-tracing (glucagon-β-gal) mice were used to investigate the transdifferentiation. The α-to-β-cell transdifferentiation is evaluated by double staining of both glucagon and insulin. They observed the double-positive cells. By the linage tracing animal model, this transdifferentiation is further confirmed by using α cell lineage-tracing. With the dapagliflozin therapy, some insulin-positive cells were located in the duct compartment or even co-localized with duct cell markers, suggestive of duct-derived beta cell neogenesis. They also performed *in vitro* study by the cultured primary rodent islets and αTC1.9 cells. Their study indicated that dapagliflozin upregulated the expression of pancreatic endocrine progenitor and β-cell specific markers as Pdx-1, Pcsk1 and GLP-1 in αTC1.9 cells.

Furthermore, in a rat model of streptozotocin-induced diabetes, physical exercise has demonstrated a protective effect against cytokine-induced pancreatic β-cell death and has been shown to promote the transdifferentiation of non-β pancreatic cells into β-cells (71). For the T1D mouse model, physical exercise could induce β-cell regeneration, demonstrated by increased proliferation and regeneration markers (Ki67 and PCNA), in islets of trained mice. The proliferated β-cells are located at both the central and the peripheral regions of the islets, accompanied by the increase in the percentage of α- and δ-cells, increase in proliferation and Pax4 labelling in peripheral regions, suggesting that both β-cell regeneration and transdifferentiation from α-cell. Meanwhile, the double positive staining of both insulin and glucagon were observed, suggesting the islet cell transdifferentiation. Moreover, there also exist the β-cell to α-cells transdifferentiation, and it has been reported that liraglutide and sitagliptin could reverse this transdifferentiation in diabetes (72). In this study, the authors investigated the lineage tracing of beta-cells in transgenic Ins1 Cre/+/Rosa26-eYFP mice. They observed that liraglutide imparted benefits on β-to α-cell transdifferentiation in hydrocortisone-induced diabetic mice. Induction of diabetes by either STZ or high fat diet, regardless of pathogenesis, led to increased numbers of β-cells losing their identity, as well as decreased expression of Pdx1 within β-cells. In these two animal models (STZ and high-fat diet), liraglutide could counter the detrimental islet cell transitioning effects. These findings highlight a range of therapeutic interventions capable of inducing α-to-β-cell transdifferentiation, offering novel approaches for the treatment of diabetes.

## 5 Challenges and prospects for the clinical application of transdifferentiation of pancreatic α-cells into β-cells

Research on the transdifferentiation of pancreatic islet α-cells into β-cells has made significant progress but still faces numerous challenges, particularly in clinical translation. One major obstacle is the inefficiency of current transdifferentiation methods, such as gene editing, transcription factor reprogramming, and drug induction, which often yield suboptimal results. Improving this efficiency necessitates a deeper understanding of the molecular mechanisms driving transdifferentiation, including strategies to overcome the intrinsic properties of α-cells and stabilize their transition into β-cell-like phenotypes. Another critical challenge is the stability of transdifferentiated cells. Even when α-cells are successfully converted into β-cell-like cells, ensuring their long-term survival and maintaining their function *in vivo* remain formidable tasks. These cells may undergo phenotypic regression, fail to fully replicate β-cell functions, or lose functionality over time due to mutations, aging, or environmental factors. Finally, the safety and efficacy of clinical applications are not yet guaranteed. Transdifferentiation processes may result in unintended gene mutations or incomplete differentiation, raising concerns about tumorigenesis. Additionally, ensuring the long-term stability and functionality of transdifferentiated cells while avoiding immune rejection requires further validation. Addressing these challenges will require a more refined understanding of the molecular pathways involved, the development of more efficient and precise induction techniques, and rigorous preclinical studies to ensure safety and efficacy for eventual clinical application.

Future research should prioritize addressing the aforementioned challenges. First, the molecular mechanisms underlying transdifferentiation, including the roles of key transcription factors and epigenetic regulation, must be further elucidated to enhance transdifferentiation efficiency. Different islet cell types may require tailored induction conditions or specific combinations of factors to achieve optimal results. Additionally, applying more precise gene editing techniques or utilizing small-molecule drugs for modulation could significantly improve the stability of transdifferentiated cells. Moreover, comprehensive preclinical studies in animal models are essential to evaluate the long-term survival, functionality, and potential side effects of transdifferentiated cells. Rigorous safety testing of gene editing technologies, such as CRISPR-Cas9, is necessary to ensure both efficacy and safety during the transdifferentiation process. With continued advancements in these areas, islet α-cell transdifferentiation holds promise as a novel and effective strategy for diabetes treatment. This approach could be particularly beneficial for patients with type 1 diabetes or those with type 2 diabetes experiencing severe β-cell failure and requiring alternative therapeutic options.

## 6 Summary

This review summarizes the fundamental mechanisms, research progress, and potential applications of transdifferentiation of pancreatic islet α-cells into β-cells for diabetes treatment. Pancreatic α-cells and β-cells play pivotal roles in maintaining blood glucose stability, with β-cells responsible for insulin secretion and α-cells producing glucagon. Recent research highlights the potential of α-cells to transdifferentiate into β-cells, particularly in diabetic patients with significant β-cell loss, offering a novel therapeutic strategy for diabetes management. By describing the essential functions and characteristics of these islet cells, we emphasize their interplay in regulating glucose homeostasis. Then, we summarized the molecular mechanisms underlying α-cell to β-cell transdifferentiation, detailing the roles of key transcription factors (e.g., *Arx*, *Pdx1*, *MafA*, *Nkx6.1*) and the distinct phases of the process, including α-cell proliferation, transdifferentiation, and stabilization of β-cell identity.

In terms of clinical applications, the transdifferentiation of α-cells into β-cells offers a promising avenue for diabetes therapy by partially restoring β-cell function and mitigating diabetic symptoms. However, significant challenges remain, such as the inefficiency of transdifferentiation, instability of transdifferentiated cells, and long-term safety concerns. We underscore the importance of advancing research to address these barriers by deepening the understanding of molecular mechanisms, enhancing cell stability, overcoming immune rejection, and promoting clinical translation. With the ongoing development of science and technology, this transdifferentiation has the potential to shed new light for the treatments for diabetes.
